# Determinants of exclusive breastfeeding in Nigeria

**DOI:** 10.1186/1471-2393-11-2

**Published:** 2011-01-11

**Authors:** Kingsley E Agho, Michael J Dibley, Justice I Odiase, Sunday M Ogbonmwan

**Affiliations:** 1 School of Medicine, the University of Western Sydney, NSW, Australia; 2 Sydney School of Public Health, the University of Sydney, NSW, Australia; 3 Department of Mathematics, the University of Benin, Benin, Nigeria

## Abstract

**Background:**

Exclusive breast feeding (EBF) has important protective effects on the survival of infants and decreases risk for many early-life diseases. The purpose of this study was to assess the factors associated with EBF in Nigeria.

**Methods:**

Data on 658 children less than 6 months of age were obtained from the Nigeria Demographic and Health Survey (NDHS) 2003. The 2003 NDHS was a multi-stage cluster sample survey of 7864 households. EBF rates were examined against a set of individual, household and community level variables using a backward stepwise multilevel logistic regression method.

**Results:**

The average EBF rate among infants younger than 6 months of age was 16.4% (95%CI: 12.6%-21.1%) but was only 7.1% in infants in their fifth month of age. After adjusting for potential confounders, multivariate analyses revealed that the odds of EBF were higher in rich (Adjusted Odds Ratios (AOR) = 1.15, CI = 0.28-6.69) and middle level (AOR = 2.45, CI = 1.06-5.68) households than poor households. Increasing infant age was associated with significantly less EBF (AOR = 0.65, 95%CI: 0.51-0.82). Mothers who had four or more antenatal visits were significantly more likely to engage in EBF (AOR = 2.70, 95%CI = 1.04-7.01). Female infants were more likely to be exclusively breastfed than male infants (AOR = 2.13, 95%CI = 1.03-4.39). Mothers who lived in the North Central geopolitical region were significantly more likely to exclusively breastfeed their babies than those mothers who lived in other geopolitical regions.

**Conclusions:**

The EBF rate in Nigeria is low and falls well short of the expected levels needed to achieve a substantial reduction in child mortality. Antenatal care was strongly associated with an increased rate of EBF. Appropriate infant feeding practises are needed if Nigeria is to reach the child survival Millennium Development Goal of reducing infant mortality from about 100 deaths per 1000 live births to a target of 35 deaths per 1000 live births by the year 2015.

## Background

Exclusive breastfeeding (EBF) for the first 6 months of life improves the growth, health and survival status of newborns [1] and is one of the most natural and best forms of preventive medicine [2,3]. EBF plays a pivotal role in determining the optimal health and development of infants, and is associated with a decreased risk for many early-life diseases and conditions, including otitis media, respiratory tract infection, diarrhoea and early childhood obesity [4].

It has been estimated that EBF reduces infant mortality rates by up to 13% in low-income countries [5]. A large cohort study undertaken in rural Ghana concluded that 22% of neonatal deaths could be prevented if all infants were put to breast within the first hour of birth [6]. According to a recent analysis, suboptimum breastfeeding, especially non-EBF in the first 6 months of life, results in 1.4 million deaths and 10% of the disease burden in children younger than 5 years in low-income and middle-income countries [7].

The importance of breastfeeding as a determinant of infant nutrition, child mortality and morbidity has long been recognized and documented in the public health literature. In response to this, the Nigerian government established the Baby-Friendly Hospital Initiative (BFHI) in Benin, Enugu, Maiduguri, Lagos, Jos and Port Harcourt with the aim of providing mothers and their infants a supportive environment for breastfeeding and to promote appropriate breastfeeding practices [8], thus helping to reduce infant morbidity and mortality rates. Despite these efforts, child and infant mortality continue to be major health issues affecting Nigeria. The infant mortality rate for the most recent five-year period (1999-2003) is about 100 deaths per 1,000 live births [9] and EBF rates in Nigeria continue to fall well below the WHO/UNICEF recommendation of 90% EBF in children less than 6 months in developing countries [5,10].

The low rate of EBF in Nigeria may, in part, be due to traditional beliefs, practices and rites. For example, in Yoruba and Bini communities, EBF is considered dangerous to the health of the infant who is thought to require water to quench thirst or stop hiccoughs [11]. Further, because the majority of women deliver outside health facilities across the community [8], the BFHI strategy alone may not have a positive effect on EBF rates.

A more detailed understanding of the factors associated with EBF in Nigeria is needed to develop effective interventions to improve the rates of EBF and thus reduce infant mortality. The purpose of the present study is to use existing representative survey data to identify the factors associated with EBF in Nigeria. The secondary data analysis aims to investigate the factors associated with rates of EBF after controlling for individual, household and community characteristics.

## Methods

The present analysis was based on a publicly available datasets and the data was collected for the Nigeria Demographic and Health Survey (NDHS) 2003 [9], conducted by the National Population Commission (NPC) [12]. The NDHS is a useful and valid source of information on EBF from a nationally representative sample of households. The survey sample was selected in two stages. In the first stage, 365 clusters were selected from a list of enumeration areas developed from the 1991 population census. In the second stage, a complete listing of households was carried out in each selected cluster. A total of 7864 households were selected for the sample, of which 7327 were interviewed, yielding a response rate of 93.2%.

From the sampled households, 7620 ever-married women in the age group 15-49 (response rate 95.4%) were interviewed using a questionnaire to collect data regarding the respondent's background, maternal and childcare practices including infant feeding, reproduction and contraception. Another questionnaire was used to collect socio-demographic information for all persons usually residing in each household, as well as an inventory of household facilities and assets. Our analysis was restricted to the youngest living children aged less than 6 months, living with the respondent (ever-married women age 15-49 years), alive and the total weighted sample size was 658.

EBF rate was defined as the proportion of infants, aged less than 6 months, who received only breast milk and not other liquids or solids except for drops or syrups consisting of vitamins, minerals supplements or medicines [2,3]. The EBF rate was estimated according to the WHO recommendation definition of this key Infant and Young Child Feeding (IYCF) indicator [3] and recommends further categorisation of EBF indicator for the following age ranges 0-1, 2-3 and 4-5 months of age [3]. It should be noted that this indicator does not represent the proportion of infants who are exclusively breastfed until their sixth month of age. A wealth index was constructed from data collected in the household questionnaire, using methods recommended by the World Bank Poverty Network and United Nations Children's Fund [13], and was divided into three categories. The bottom 40% of the households was referred to as the poorest households, the next 40% as the middle-level households, and the top 20% as the richest households.

EBF indicator was expressed as a dichotomous variable with category 1 for EBF and category 0 for non-EBF. This variable was examined against a set of independent variables (individual, household and community characteristics) in order to determine the prevalence of EBF and factors associated with the rate of EBF. Data analysis was performed using the "SVY" commands of Stata version 10.0 (Stata Corp, College Station, TX, USA), which allowed for adjustments for sampling weights when estimating confidence intervals around prevalence estimate. Chi-squared tests were used to assess the significance of associations. Logistic regression was conducted using stepwise backwards generalized linear latent and mixed models (gllamm) [14] method in order to determine the factors significantly associated with the rate of EBF. The odds ratios with 95% confidence intervals were calculated in order to assess the adjusted risk of independent variables, and those with p < 0.05 were retained in the final model

## Results

### Characteristics of the sample

As summarized in Table 1, the majority of children lived in rural areas (72.7%). Approximately 56% of the mothers of the children were employed in the last 12 months, and 26.6% had completed secondary or higher level of education. Of the total births, 30.9% took place at a health care facility. The proportion of deliveries by caesarean section was relatively low (1.0%). Male and female children were nearly equally represented in the sample. About 61% of mothers had made at least one antenatal clinic visit during pregnancy, and 47.4% of the mothers did not have any post-natal check up after 41 days.

**Table 1:** Individual, household and community level characteristics and rates of EBF of children <6 months of age, Nigeria 2003 (n = 658).

Characteristic			Exclusive breastfeeding rates (<6 months, n = 658)	
	
	n	%	% 95% CI	Pvalue
*Individual level factors*				
Maternal working status (n = 657)				
Non-working	293	44.6	14.2 (9.6 - 20.4)	
Working (past 12 months)	365	55.5	18.2 (13.5 - 24.0)	0.338
Maternal education				
No education	333	50.6	11.0 (7.5 - 16.0)	
Primary	151	22.9	12.7 (7.8 - 20.0)	<0.001
Secondary and above	175	26.6	29.7 (19.9 - 41.9)	
Partner's education (n = 629)				
No education	254	40.4	10.6 (7.0 - 15.8)	
Primary	295	46.9	16.4 (11.5 - 22.7)	0.003
Secondary and above	80	12.8	30.6 (18.1 - 46.8)	
Mother's age				
15-19 years	264	40.1	15.4 (10.0 - 22.9)	
20-34 years	294	44.6	18.9 (13.7 - 25.5)	0.358
35-49 years	100	15.3	11.5 (5.7 - 22.0)	
Marital status (n = 635)				
Currently married	623	98.1	15.5 (11.7 - 20.1)	
Formerly married (div/sep/widow)	12	1.9	35.3 (13.2 - 66.1)	0.050
Birth order				
First-born	144	21.9	23.6 (13.2 - 38.4)	
2nd-4th	296	44.9	16.5 (11.8 - 22.7)	0.104
5 or more	219	33.2	11.4 (7.4 - 17.1)	
Preceding birth interval				
No previous birth	144	21.9	23.6 (13.2 - 38.4)	
<24 months	73	11.1	25.8 (13.6 - 43.6)	0.044
≥24 months	441	67.1	12.5 (9.3 - 16.6)	
Sex of baby				
Male	345	52.5	15.2 (10.5 - 21.3)	
Female	313	47.5	17.7 (13.2 - 23.3)	0.409
Age of child (months)				
0-2.9	303	46.1	22.2 (16.5-29.1)	
3-5.9	355	53.9	10.7 (7.6 - 14.9)	<0.001
Place of delivery				
Home	455	69.1	12.5 (8.8 - 17.5)	
Health facility	203	30.9	25.0 (17.5 - 34.4)	0.003
Mode of delivery (n = 650)				
non-caesarean	643	99.0	15.8 (12.0 - 20.6)	
Caesarean section	7	1.0	26.6 (7.2 - 62.7)	0.104
Type of delivery assistance (n = 565)				
Health professional	221	39.1	23.3 (16.8 - 31.4)	
Traditional birth attendance	114	20.2	12.8 (6.5 - 23.7)	0.045
Other	230	40.7	14.3 (8.7 - 22.7)	
Antenatal Clinic visits (n = 644)				
None	251	39.1	6.0 (3.5 - 10.2)	
1-3.	98	15.2	19.8 (11.5 - 31.8)	<0.001
4+	295	45.8	23.5 (17.2 - 31.2)	
Timing of postnatal check-up				
0-2 days	105	16.1	10.5 (5.1-10.2)	
3-41 days	21	3.2	6.7 (1.5 - 25.0)	<0.001
Did not receive postnatal check-up^1^	314	47.4	25.1 (18.7 - 32.9)	
Missing	218	33.2	6.7 (4.2 - 10.6)	
Mother's BMI (n = 644)				
< = 18.5	70	10.9	9.4 (4.5 - 18.7)	
>18.5	573	89.1	17.6 (13.3 - 22.9)	0.159
Size of baby (n = 652)				
Small	108	16.6	9.9 (4.5 - 20.4)	
Average	289	44.4	16.6 (12.4 - 21.9)	
Large	255	39.0	18.5 (12.2 - 27.1)	0.364
Mothers Literacy (n = 648)				
Can't read	405	62.6	10.5 (7.3 - 14.7)	
Read part/whole sentences	243	37.5	25.7 (17.9 - 35.3)	<0.001
*Household level factors*				
Wealth Index				
Poor	285	43.4	8.3 (5.4 - 12.6)	
Middle	248	37.7	20.6 (14.5 - 28.5)	
Rich	124	18.9	26.4 (16.6 - 39.2)	<0.001
Decisions women have final say				
None	310	47.1	16.5 (10.7 - 24.7)	
1-2.	212	32.2	13.8 (8.2 - 22.4)	0.725
3-4.	84	12.8	19.1 (11.6 - 29.9)	
5	51	7.8	21.5 (9.9 - 40.7)	
*Community level factors*				
Residence				
Urban	180	27.3	20.7 (14.7 - 28.4)	
Rural	478	72.7	14.7 (10.2 - 20.9)	0.179
Geographical region				
North Central	89	13.5	40.5 (29.7 - 52.5)	
North East	149	22.6	11.5 (5.3 - 23.0)	
North West	233	35.5	7.4 (4.4 - 12.3)	<0.001
South East	35	5.3	9.6 (3.3 - 24.9)	
South South	85	13.0	14.7 (5.5 - 33.6)	
South West	67	10.1	32.2 (19.5 - 48.1)	

Overall	658	100	16.4 (12.6 - 21.1)	NA

Weighted total count = 658 unless otherwise given in brackets, Pvalue obtained by chi-squared test

^1^includes women who received the first postnatal check-up after 41 days

NA = Not applicable

According to the mothers' perceptions, 43.9% of children were of average size at birth. The proportion of mothers who could not read a sentence was 62.6%. The majority of children were from the North West and the North East regions of the country.

#### Exclusive breast feeding (EBF) rate

**Figure 1:**
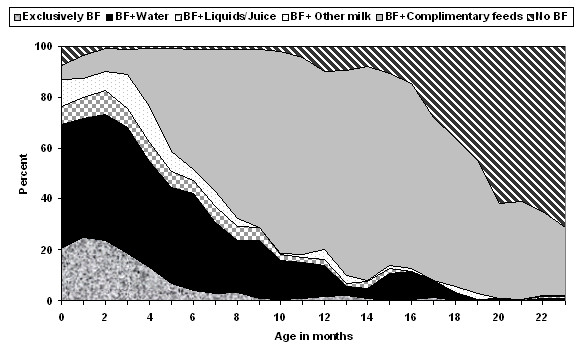
**Distribution of children by breastfeeding (BF) status, according to age, Nigeria 2003**.

Of the total sample of 658 children aged below 6 months from Nigeria, the proportion of infants who were EBF was 16.4%. The proportion of EBF infants 0 to 1 months of age was 26.1%; 18.5% at 2 to 3 months of age, 7.1% at 4 to 5 months of age and 21.9% at 0 to 3 months of age. As shown in Figure 1, the proportion of EBF infants was 20% at birth; 19% at 2 months, 13% at 4 months and further declined to about 4% at 5 months. Furthermore, Figure 1 reveals that the rate of EBF was about 4.5 fold lower than the WHO/UNICEF recommended level of 90% for EBF in children less than 6 months of age. At birth, a high proportion of the infants were being breastfed plus water (49%), or breastfed plus water-based liquid or juice (7%), breastfed plus other milk (10%), or breastfed plus complimentary foods (6%).

#### Univariate analysis

Table 1 presents the estimated percentage of infants younger than 6 months, who were EBF by selected individual, household and community characteristics. It reveals that EBF rates were significantly lower among mothers who had no education compared with those who had primary, secondary or higher education. EBF rates were also higher among women who had delivery assistance from health professionals compared to those mothers who were assisted by traditional birth attendants or untrained persons. Those mothers who had 4 or more antenatal clinics visits during pregnancy reported significantly higher EBF rates than those who made no antenatal clinic visits (23.5% vs. 6.0%).

Univariate analyses indicated a significantly lower EBF rate for infants less than 6 months among mothers who lived in rural regions (14.7%) compared to those who lived in urban regions (20.7%). Univariate analyses revealed that geopolitical region, household wealth, mother's literacy, preceding birth interval, type of delivery assistance, place of delivery, and number of antenatal clinic visits, were all significantly associated with EBF.

#### Multivariate analysis

Unadjusted and adjusted odds ratios (AOR) were calculated to determine the strength of association between independent variables and EBF (see Table 2). As expected, increasing age of the infant was associated with significantly less EBF (AOR = 0.65, 95%CI = 0.51-0.82). Mothers who had no antenatal visits during pregnancy had lower odds for EBF than those mothers who had 1 to 3 antenatal clinic visits, and 4 or more antenatal clinic visits (AOR = 2.62, 95%CI = 0.83-8.31 and AOR = 2.70, 95%CI = 1.04-7.01, respectively). Infants from the poorest households were less likely to be EBF compared to infants from middle-level (AOR = 2.45, 95%CI = 1.06-5.68) and wealthiest households (AOR = 1.15, 95%CI = 0.28-6.69).

**Table 2:** Risk factors for a child being exclusively breastfed - unadjusted and adjusted Odds Ratio (OR), Nigeria 2003

Characteristic	EBF			
	
	Unadjusted odds ratios		Adjusted 0dds ratios (AOR)	
	
	OR (95% CI)	*p*	AOR (95% CI)	*p*
Antenatal Clinic visits				
None	1.00		1.00	
1-3.	5.02 (1.55 - 16.28)	0.007	2.62 (0.83 - 8.31)	0.102
4+	4.33 (1.71 - 10.9)	0.002	2.70 (1.04 - 7.01)	0.040
Wealth Index				
Poor	1.00		1.00	
Middle	3.31 (1.41 - 7.81)	0.006	2.45 (1.06 - 5.68)	0.036
Rich	1.89 (0.48 - 7.43)	0.360	1.15 (0.28 - 6.69)	0.841
Geopolitical region				
North Central	1.00		1.00	
North East	0.19 (0.07 - 0.54)	0.002	0.19 (0.06 - 0.59)	0.004
North West	0.12 (0.04 - 0.36)	<0.001	0.16 (0.06 - 0.49)	0.001
South East	0.28 (0.05 - 1.42)	0.124	0.20 (0.03 - 1.22)	0.082
South South	0.48 (0.13 - 1.80)	0.273	0.47 (0.11 - 1.95)	0.299
South West	0.39 (0.08 - 2.04)	0.266	0.32 (0.06 - 1.83)	0.200
Child's age				
Age of child (months)	0.64 (0.53 - 0.77)	<0.001	0.65 (0.51 - 0.82)	<0.001
Sex of baby				
Male	1.00		1.00	
Female	1.99 (0.98 - 4.03)	0.057	2.13 (1.03 - 4.39)	0.041

Note: Independent variables adjusted for are: maternal working status; maternal education; partner's education; mother's age; marital status; birth order; preceding birth interval; sex of baby; child's age in month; place of delivery; mode of delivery; type of delivery assistance; timing of postnatal check-up; mother's Body mass index (BMI); size of baby; mothers literacy; wealth Index; decisions women have final say, residence and geopolitical region

Compared to the North Central region, mothers who lived in the following geopolitical regions of Nigeria were significantly less likely to EBF their babies: North East (AOR = 0.19, 95%CI = 0.06-0.59); North West (AOR = 0.16, 95%CI = 0.06-0.49); South East (AOR = 0.20, 95%CI = 0.03-1.22), South South (AOR = 0.47, 95%CI = 0.11-1.95) and South West (AOR = 0.32, 95%CI = 0.06-1.83). Female infants were more likely (AOR = 2.13, 95%CI = 1.03-4.39) to be exclusively breastfed than male infants.

## Discussion

We found a very low rate of EBF in Nigeria that needs improvement in order to gain the full health benefits of breastfeeding. The levels are far below the program target of 90% of women exclusively breastfeeding their infants in the first 6 months of life, which is associated with a reduction of 10% of under-five deaths [5]. The key factors that were associated with higher rates of EBF included antenatal clinic visits, household wealth and gender. Also of importance were the types of addition liquids and foods given to young infants that resulted in the low EBF rates.

Nearly 50% of infants less than 3 months of age received breast milk and water (see Figure 1) and this remained high at about 40% at 6 months of age. These findings may reflect the cultural practice of giving water plus breast milk by some communities in Nigeria to quench the child's thirst [11]. However, the practice of breastfeeding plus giving water may increase mortality rates in Nigeria due to contaminated water and poor sanitation [15]. Our findings on the frequent use of water with breast feeding suggest substantial improvement in EBF rates could be achieved with simple focused messages about not giving water to these young infants.

Mothers from socioeconomically privileged groups were more likely to EBF their babies than those from lower socioeconomic class. Our results indicated a correlation between household wealth and level of education, with 13.7% of mothers practising EBF from poor households with low education levels compared to those from rich households with secondary education or higher (26.1%). However, the reported prevalence of EBF among educated Nigerian mothers is relatively low compared to countries like Nepal, Bangladesh and India [16-18]. The reported low prevalence among educated Nigerian mothers may be attributed to current economic challenges in Nigerian, where mothers may be forced to return to full time work causing a shorter duration of breastfeeding [8]. In our analysis mothers who visited health professionals, or made 4 or more antenatal clinic visits were significantly more likely to exclusively breastfeed their babies, and these findings suggests that appropriate message about breastfeeding are being delivered by antenatal care staff. Furthermore, these findings were similar to those reported in earlier studies [19,20]. But our findings contradict a randomized controlled trial (RCT) [21] in Italian women, which concluded that using health workers alone to give early support for EBF was ineffective.

The odds of mothers practising EBF were relatively low in all geopolitical regions but much lower in North East and North West geopolitical regions. Mothers who lived in these regions were less likely to practice EBF than those who lived in the North Central geopolitical regions. These findings are similar to those reported from countries in Southeast Asia where the rate of EBF was significantly associated with particular sub-national geographical area [20].

Multivariate multilevel binary logistic regression analysis revealed that the following factors were significantly associated with EBF after controlling for confounders: (a) decreased child age in months; (b) geopolitical region; (c) antenatal clinic visits; (d) household wealth, and (f) gender. A recent study in East and Southeast Asia found that region, household wealth and sex of the baby were positively associated with EBF [8,19,22]. Evidence from Edo and Oyo State in Nigeria also supported the finding that younger age, sex of the baby and antenatal contacts with health centres predicted improved EBF [8,19,22].

In keeping with the Millennium Development Goals, countries in the developing world have committed to reduce under 5 mortality rates by two thirds between 1990 and 2015 [23]. The efforts of the world community to reduce high infant and child mortality and their associated factors including malnutrition and not breastfeeding exclusively have succeeded in some parts of the world [24] but have remained unsatisfactory in Nigeria. Of greater concern, is that EBF in Nigeria is in decline. In the NDHS of 1999, 22% of infants aged 0-6 months were exclusively breastfed, however, this decline to 17.2% in the NDHS of 2003 [9].

The main strengths of the study are the nationally representative sample, comprehensive data on standard infant feeding indicators, appropriate adjustment for sampling design, including sampling weight and a very high response rate (95.4%) to the survey interview. The EBF indicator examined was based on the standard definitions formulated by WHO [3].

A limitation of this paper is the potential for recall bias because mothers had to remember how the child was fed in the preceding day and the capacity to recall food might vary by key factors we examined in the analysis such as maternal educational status. The definition of EBF was based on the 24 hour recall, and the day to day variability in food intake might lead to an over estimation of EBF. Despite these limitations, the findings from this study contribute to our understanding of the factors associated with EBF rate in Nigeria.

## Conclusions

EBF rates in Nigeria are amongst the lowest in the world, and even compare poorly with other neighbouring countries in the region - Nigeria lags behind Ghana (53.4%), Republic of Benin (43.1%) and Cameroon (23.5%) [25-27]. A substantial improvement of EBF can be achieved in Nigeria by avoiding the practice of mothers giving water to their babies in addition to breast milk. EBF promotion programmes should target all mothers, but with special focus on poor and illiterate families, mothers who delivered at home and mothers who have had no antenatal clinic visits. In addition, further research is required to describe the feeding patterns and dietary intake related to complementary feeding, and their effects on children's growth. Finally, intervention studies, including peer counselling using cluster-randomised controlled trials, are needed to improve EBF among mothers and those having their first baby in Nigeria.

## Competing interests

The authors declare that they have no competing interests.

## Authors' contributions

KA wrote the manuscript. All authors made contributions to the interpretation of results and revised the manuscript for important intellectual content. All authors read and approved the final version

## Pre-publication history

The pre-publication history for this paper can be accessed here:

http://www.biomedcentral.com/1471-2393/11/2/prepub
